# Commentary: Sound-making actions lead to immediate plastic changes of neuromagnetic evoked responses and induced β-band oscillations during perception

**DOI:** 10.3389/fnins.2018.00050

**Published:** 2018-02-07

**Authors:** Rebecca W. Gelding, Yanan Sun

**Affiliations:** ^1^Centre of Excellence in Cognition and its Disorders (ARC), Sydney, Australia; ^2^Department of Cognitive Science, Macquarie University, Sydney, Australia; ^3^Department of Psychology, Macquarie University, Sydney, Australia

**Keywords:** auditory perception, motor activity, beta band oscillations, neuroplasicity, P2 component, passive listening

Learning an action that is coupled to a sound, changes the brain's response to that sound. This is seen in experienced musicians (Pantev and Herholz, 2011) and expert dancers (Orgs et al., 2008), however changes occur even in novice musicians after 6 weeks of piano practice (Herholz et al., 2016), or overnight, after perceptual training (Alain et al., 2015). Ross et al. (2017) investigated whether neuroplastic changes in perception after a learned action-sound association, occur immediately.

Ross et al. (2017) used magnetoencephalographic (MEG) recordings to measure auditory evoked and oscillatory responses, while participants passively listened to an unfamiliar instrument (Tibetan singing bowl struck with wooden mallet) for 24 min, then actively made the sounds themselves for 30 min, followed by another passive listening period of 24 min. A control group had identical listening blocks, but during the sound-making period, initiated the sound of the bell through a button press on keypad rather than striking the bowl.

By comparing the passive listening trials before and after sound making, Ross et al. found: (1) increased bilateral auditory evoked P2 response, (2) greater event-related desynchronization (ERD) in the β-band in right auditory and left sensorimotor sources, (3) increased functional connectivity in the θ-band between the left sensorimotor source and bilateral auditory sources. These results confirm that auditory perceptual learning can elicit immediate functional neuroplasticity. Given these changes are seen in the experimental but not the control group, Ross et al. concluded that learning the specific action of hitting the singing bowl with a mallet induced more rapid and efficient establishment of an action-sound association a common key press (Ross et al., 2017).

The immediate increase in P2 amplitude, within 1 h of the experimental procedure, stands in contrast to previous research, that observed no immediate increases in P2 amplitude (Alain et al., 2007). The main difference between the two studies being that Alain et al. (2007) applied learning-by-listening tasks with no motor-training. Given the P2 increase in Ross et al. (2017) is not seen in the control group, this suggests that these neuroplastic changes depend critically on the nature of training. Striking a singing bowl is more efficient in establishing the specific association between perception and action, than pressing a key. Therefore, the perception-action association in the experimental group enhanced object representation for perception more rapidly than for the control group.

Alternative control conditions examining alternative motor and auditory input could also explore how neuroplastic changes depend on the nature of training. Motor timing has been shown to be more accurate when more motor effectors are used [e.g., hand tapping with a stick vs. finger tapping Manning et al., 2017] suggesting that motor engagement using the hand and the finger are not entirely comparable. They have different degrees of freedom in effector movement (Latash, 2014) and different levels of tactile feedback as an action outcome (Wing et al., 2010). This may explain why greater immediate changes were seen in Ross et al.'s experimental group than the control group. Future studies could use a control condition with similar motor activity and auditory stimuli to experimental condition (e.g., an electronic drum pad, with the sound of the singing bowl played through earphones). We predict the proposed control condition would show weaker object representation, with neural responses in between the current experimental and control groups, and thus could be used to investigate how the strength of the action-perception association influences brain activity.

Additionally, the authors had one subject complete the task with sand in the bowl to gain a measure of motor action in silence, but future studies could extend this. By instructing a group of participants explicitly to imagine the sound of the singing bowl as they strike a sand filled bowl in silence, the same action would be required, but the auditory stimuli would be internally generated rather than perceived. The generation of musical imagery activates both motor and auditory cortices (Zatorre and Halpern, 2005), and engagement of mental imagery during practice has been shown to be beneficial in learning an instrument (Pascual-Leone, 2003). Accenting a tone, either through imagination or through increasing the volume, both increase the amount of β-ERD after a tone (Fujioka et al., 2015). Therefore, we would predict that for participants with sufficient imagery strength, the action-perception representation could still be established in silence. Evidence for such a representation could be seen in greater auditory β-band ERD after a tone, during post-training listening.

While β-band is historically understood as a sensorimotor rhythm (Cheyne, 2013), studies have shown a role for the β-band for temporal prediction, in the auditory cortex (Fujioka et al., 2012; Arnal et al., 2015). It has been posited that music cognition is dynamically embodied, with motor-action and perception representations actively interacting (Maes et al., 2014), and that the β-band is an “open-line” of communication between the auditory and sensorimotor regions (Tang et al., 2016). However, a causal link in the β-band between the motor and sensory regions has not yet been established (Arnal and Giraud, 2012). The functional connectivity analysis of Ross et al. shows increased correlational connectivity between eft sensorimotor and bilateral auditory regions after training. Yet, effective connectivity approaches such as Granger causality (Arnal and Giraud, 2012; Friston et al., 2013), could clarify the causation of connectivity, confirming whether sensorimotor β-band activity predicts auditory activity or vice versa. This could contribute to larger debate on embodied cognition and the role of motor regions in prediction of sensory inputs (Clark, 2013).

This paper adds to the growing evidence of the role of auditory-motor engagement in musical perception, and once again shows the effectiveness of music as a stimulus through which to investigate neuroplasticity within auditory and motor domains (Zatorre et al., 2007; Chen et al., 2008). It is the first to show immediate effects of motor learning on music perception, particularly in the time window of 200–350 ms after a tone is heard. What remains unknown is how long lasting these immediate effects are, and how they may differ from longer-term effects, acquired through expertise.

## Author contributions

All authors listed have made a substantial, direct and intellectual contribution to the work, and approved it for publication.

### Conflict of interest statement

The authors declare that the research was conducted in the absence of any commercial or financial relationships that could be construed as a potential conflict of interest.
